# Microbial Degradation of Acetamiprid by *Ochrobactrum* sp. D-12 Isolated from Contaminated Soil

**DOI:** 10.1371/journal.pone.0082603

**Published:** 2013-12-27

**Authors:** Guangli Wang, Xiao Chen, Wenlong Yue, Hui Zhang, Feng Li, Minghua Xiong

**Affiliations:** College of Life Sciences, Huaibei Normal University, Huaibei, People's Republic of China; University of Kansas, United States of America

## Abstract

Neonicotinoid insecticides are one of the most important commercial insecticides used worldwide. The potential toxicity of the residues present in environment to humans has received considerable attention. In this study, a novel *Ochrobactrum* sp. strain D-12 capable of using acetamiprid as the sole carbon source as well as energy, nitrogen source for growth was isolated and identified from polluted agricultural soil. Strain D-12 was able to completely degrade acetamiprid with initial concentrations of 0–3000 mg·L^−1^ within 48 h. Haldane inhibition model was used to fit the special degradation rate at different initial concentrations, and the parameters *q*_max_, *K*_s_ and *K*_i_ were determined to be 0.6394 (6 h)^−1^, 50.96 mg·L^−1^ and 1879 mg·L^−1^, respectively. The strain was found highly effective in degrading acetamiprid over a wide range of temperatures (25–35°C) and pH (6–8). The effects of co-substrates on the degradation efficiency of acetamiprid were investigated. The results indicated that exogenously supplied glucose and ammonium chloride could slightly enhance the biodegradation efficiency, but even more addition of glucose or ammonium chloride delayed the biodegradation. In addition, one metabolic intermediate identified as *N*-methyl-(6-chloro-3-pyridyl)methylamine formed during the degradation of acetamiprid mediated by strain D-12 was captured by LC-MS, allowing a degradation pathway for acetamiprid to be proposed. This study suggests the bacterium could be a promising candidate for remediation of environments affected by acetamiprid.

## Introduction

Neonicotinoid insecticides, which are one of the most important classes of commercial insecticides worldwide, are systemic in plants and animals and are used to manage crop pests and control fleas on cats and dogs [1]–[4]. Acetamiprid, a chloropyridinyl neonicotinoid, was considered to be a favorable choice for controlling those pests that are severely resistant to organophosphorus, urethane and synthetic pyrethroid pesticides, so it is regarded by EPA as an important substitute of organophosphorus pesticides [5]. Owing to its broad insecticidal spectrum and relatively low acute and chronic mammalian toxicity, acetamiprid is used widely in crop protection [24],[25]. Though the half life of acetamiprid in field was reported to be about 2.8–14 days [6], the risk of its ambient pollution, principally in water, is still present [7]. Acetamiprid exposure has been proven to have adverse effect on greenhouse workers spraying acetamiprid [8], soil microorganisms [25] and beneficial insects [24]. In recent years, acetamiprid residues in crops are receiving considerable attention due to their potential toxicity to humans [9], [10], and methods for the biotransformation of neonicotinoids are being actively researched. Microbial degradation is considered as an efficient “green” solution to eliminate environmental polluting chemicals [11], [12]. Acetamiprid metabolism in microorganisms has been studied in *Stenotrophomonas* sp. THZ-XP, *Rhodotorula mucilaginosa* IM-2, *Stenotrophomonas maltophilia* CGMCC 1.1788, *Pseudomonas* sp. FH2, *Phanerochaete sordida* YK-624 and *Pigmentiphaga* sp. strain AAP-1 [4],[13]–[19].

In this report, a highly effective acetamiprid-degrading strain, designated D-12, was isolated from polluted agricultural soil and identified as *Ochrobactrum* sp. Strain D-12 was able to use acetamiprid as the sole source of carbon and nitrogen for growth and completely degraded 300 mg·L^−1^ acetamiprid within 6 h. A kinetic model of acetamiprid degradation and transformation was proposed. Effects of several parameters including pH, temperature, initial substrate concentration and second carbon/nitrogen source as co-substrate on the biodegradation of acetamiprid by the isolated strain were investigated. Moreover, the pathway of acetamiprid biodegradation mediated by strain D-12 is proposed. This paper highlights a significant potential use of pure cultures of microbial cells for the cleanup of acetamiprid-contaminated soil.

## Materials and Methods

### Chemicals and Media

Analytical grade acetamiprid (purity, 99%), purchased from Wako Pure Chemical Industries (Osaka, Japan), was used as a standard. Acetamiprid samples (>97% purity) were purchased from Dongfeng Pesticides Factory (Shanghai, China). Chromatographic grade acetonitrile and acetic acid were purchased from Sigma-Aldrich (St. Louis, USA). Molecular biology reagents were purchased from TaKaRa Biotechnology Co., Ltd (Dalian). All other chemicals were of the highest grade that was commercially available. Luria-Bertani (LB) medium (10.0 g NaCl, 10.0 g peptone and 5.0 g yeast extract per litre water, pH 7.0) and mineral salts medium (MSM) (1.5 g K_2_HPO_4_, 0.5 g KH_2_PO_4_, 0.2 g MgSO_4_·7H_2_O, 1.0 g NaCl per litre water, pH 7.0) were utilised in this study. For solid medium, agar powder was added at a concentration of 1.6%. When necessary, acetamiprid was added to the media at an appropriate concentration. All media used in this study were prepared using Milli-Q water (>18.2 MΩ) and sterilised by autoclaving at 121°C for 25 min.

### Isolation and identification of bacteria

A soil sample was collected from an agricultural field, which had been exposed to acetamiprid for more than 10 years, in the city of Yancheng, China. Enrichment and isolation of degrading bacterial isolates were conducted as described in detail previously [18]. The ability of isolates to degrade acetamiprid was determined by HPLC following the protocol described below. One pure isolate designated D-12 showing the highest degradation activity was selected for further study.

Strain D-12 was characterized based on its morphological, physiological and biochemical properties [20] and genetic analysis based on 16S rRNA gene sequence. The cell morphology was examined by light microscopy (BH-2, Olympus, Japan) and transmission electron microscopy (H-7650, Hitachi High-Technologies Corp., Japan), using cells from an exponentially growing culture. The genomic DNA of D-12 strain was extracted by high-salt precipitation [21]. Pure cultures were phylogenetically characterized using 16S rRNA gene sequencing. Two PCR primers 27F/1492R were designed to amplify the 16S rRNA gene [22]. The 1389 bp 16S rRNA gene sequence was compared to sequences in GenBank using BLAST program. Multiple sequence alignment was carried out using Clustal X 1.8.3 with the default settings. For further phylogenetic analysis, MEGA version 4.0 software [23] was used. Distances were calculated using the Kimura two-parameter distance model. Unrooted trees were built using the Neighbor Joining method. The date set was bootstrapped 1000 times.

### Acetamiprid degradation experiments

The isolated strain was grown on LB medium for 16 h at 30°C on a rotary shaker (160 rpm). Then cells were harvested and washed three times with a 0.02 mol·L^−1^ phosphate buffer (pH 7.0). The washed cells were re-suspended in the same buffer, resulting in a cell suspension with an OD_600_ of 1.0. In addition, a stock solution of acetamiprid (4,000 mg·L^−1^) was prepared by dissolving the acetamiprid in MSM. After transferring appropriate volume of this stock solution to a 250 mL sterile flask, 100 mL of sterilized MSM was added. 1 mL of the prepared cell suspension was then inoculated into the medium for biodegradation assessment. All tests were conducted in triplicate.

To assess the effects of pH, temperature and initial substrate concentration on acetamiprid degradation, incubation temperature (15, 20, 25, 30, 35, 40 and 45°C), initial acetamiprid concentration (250, 500, 1000, 1500, 2000, 2500 and 3000 mg·L^−1^) and media pH (4.0–10.0, in increments of 1.0 pH units) on biodegradation of acetamiprid by strain D-12 were studied.

To study the effect of second carbon or nitrogen sources as co-substrate on the degradation of acetamiprid, different dosages (100, 200, 300, 400, and 500 mg·L^−1^) of glucose as a second carbon source as well as different dosages (300, 600 and 900 mg·L^−1^) of ammonium chloride as a nitrogen source as well as different dosage of glucose (100, 200, 300, 400 and 500 mg·L^−1^) as a second carbon source with the presence of 300 mg·L^−1^ of ammonium chloride as a nitrogen source was respectively added into flasks which contained 100 mL of MSM medium, 5 mL of strain D-12 culture and 300 mg·L^−1^ of acetamiprid. Acetamiprid added as sole carbon and nitrogen source was set as control. Each treatment was set in triplicate.

### Chemical Analysis

Cell growth was monitored by measuring the optical density of culture samples at 600 nm (OD_600_). Non-inoculated medium served as control. For acetamiprid extraction from liquid culture, 5 mL of sample collected from the medium was extracted with 10 mL of dichloromethane. After shaking for 10 min, the dichloromethane phase was dried over anhydrous Na_2_SO_4_, and the solvent was removed using a stream of nitrogen at room temperature. The residues were dissolved in 200 µL of acetonitrile. Samples in acetonitrile were then filtered through a 0.22 µm Millipore membrane filiter. An aliquot of the solution (20 µL) was injected into an HPLC system for detection.

The concentration of acetamiprid was determined by HPLC using a Zorbax C-18 ODS Spherex column (250 mm*4.6 mm). The mobile phase was 65% (volume) water and 35% (volume) acetonitrile as well as 0.01% acetic acid at a flow rate of 1 mL·min-1. The eluate was monitored by measuring the A242 with a Waters 2487 Dual Wavelength Absorbance Detector, and the injection volume was 20 µL. Recovery efficiency of the stated method was evaluated at the concentrations of 10, 30, 50 and 70 mg·L^−1^ acetamiprid that appended in MSM.

The metabolites produced during acetamiprid degradation were purified using thin-layer chromatography (TLC) by concentrating the extract on a pre-coated silica-gel TLC plate (silica G, 20×20 cm, 0.25 mm thickness) with chloroform-methanol solution (20∶1 by volume). The collected metabolite was dissolved in acetonitrile and centrifuged at 10,000 g to remove the silica. The organic solvents were then removed and the residue was dried under vacuum condition.

The purified metabolites were analyzed by standard MS, ionized by electrospray with a positive polarity, and scanned in the normal mass. Characteristic fragment ions were detected with second-order MS.

The MS apparatus was an LC-MSD-Trap-SL system equipped with an electrospray ionization source and was operated in the positive polarity mode. The ES-MS interface was operated using a gas temperature of 35°C and a drying gas flow of 9.0 L min^−1^. The nebulizer nitrogen gas pressure was 45 psi. Full scan signals were recorded within the m/z range from 50 m/z to 600 m/z. For LC-MS, the spray voltage was 7.0 kV. The sheath and auxiliary gases were nitrogen. The sheath and auxiliary gases were adjusted to 65 and 10 arbitrary units, respectively. Auto Gain Control mode was used to optimize the injection time.

### Data Analysis

Results were also assessed by analysis of variance (ANOVA) and statistical analyses were performed on three replicates of data obtained from each treatment. The significance (*P*<0.05) of differences was treated statistically by single factor analysis of variance using SPSS software packages.

### Ethics statement

The location that we collected samples was a very common field, so it did not need specific permission and involve any endangered or protected species. In order to avoid being influenced by plant diseases and insect pests, acetamiprid was used discontinued ten years ago.

## Results and Discussion

### Evaluation of the analytical method for acetamiprid determination

The recovery efficiency of acetamiprid in MSM are arranging from 87% to 104%. The evaluation results displayed that the analytical method applied in this study well satisfied the requirement of the pesticide analysis standard (Table 1; satisfy scopes are from 80% to 120%). The LOD (limit of detection) and LOQ (limit of quantification) of the method were 1.0×10^−10^ g and 0.002 mg·L^−1^, respectively.

**Table 1:** Recovery efficiency and regression equation determined by different concentrations of AAP for the analytical method evaluation.

Append concentration (mg•L^−1^)	Detected concentration	Recovery efficiency (%)	Regression equation
10	10.44±0.53	104	
30	28.12±1.45	94	y = 12.224×+1.0760
50	43.34±0.32	87	R^2^ = 0.9982
70	67.35±2.15	91	

Regression equation was determined by HPLC using different concentrations of standard AAP; y, AAP concentration; x, the peak area of HPLC; R^2^, correlation coefficient of the regression equation.

### Isolation and identification of the acetamiprid-degrading strain D-12

**Figure 1:**
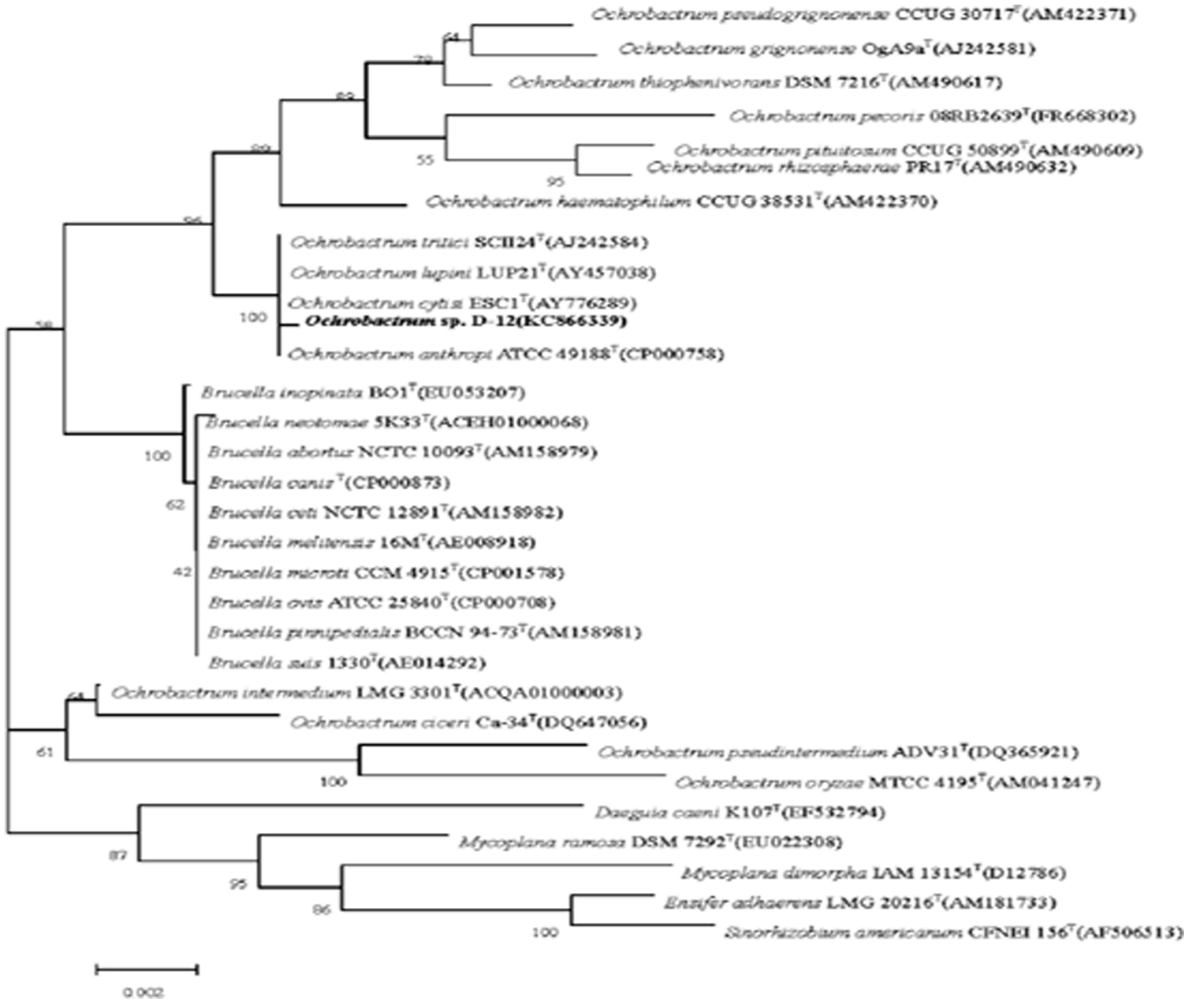
Phylogenetic tree constructed by the neighbor-joining method based on 16S rRNA gene sequences of D-12 and type strains of *Ochrobactrum* species. Bootstrap values, expressed as percentages of 1,000 replications, are given at branching points. Bar, 0.002 nucleotide substitutions per nucleotide position.

Acetamiprid is a member of the neonicotinoid group of insecticides commonly used against wide range of insect pests. Owing to its broad insecticidal spectrum and relatively low acute and chronic mammalian toxicity, acetamiprid is used widely in crop protection [24], [25], therefore, it is probable that several bacteria have adapted to this acetamiprid-contaminated environment. A pure bacterial strain that could grow by using acetamiprid as the sole source of carbon and nitrogen was obtained from contaminated soil. The isolated strain was found to be aerobic, non-spore-forming, Gram-negative rods and motile with a polar flagellum (Fig. S1). Colonies of strain D-12 on LB agar were white to beige, mucoid with entire edges and 2–3 mm in diameter within 24 h at 30°C. Strain D-12 was positive for oxidase, catalase, β-galactosidase, α-Glucosidase, Lipase C14 and Nitrate reduction and negative for indole and urease. It could hydrolyze Aesculin. Strain D-12 could grow in LB in the presence of 7% NaCl (w/v), and the optimal growth was at 1–2% NaCl (w/v). Growth occurs at temperature of 4–42°C with an optimum at 28–30°C. Growth occurs at pH 6.0–9.0, with an optimum at pH 7.0–7.5. Strain D-12 could use glucose, D-Mannose, L-Arabinose, D-Turanose, L-Lyxose, Sodium acetate as sole carbon sources for growth but failed to utilize Citrate and L-Arabinose (Table 2). Phylogenetic analysis of the 16S rRNA gene sequences (Fig. 1) revealed strain D-12 clustered with members of the genus *Ochrobactrum*, and had 100% sequences similarity with *Ochrobactrum tritici* SCII24^T^ (AJ242584), *Ochrobactrum lupini* LUP21^T^ (AY457038), *Ochrobactrum anthropi* ATCC 49188^T^ (CP000758) and *Ochrobactrum cytisi* ESC1^T^ (AY776289). Members of this genus have become the focus of some studies because of its versatile biodegradability. A few of strains from *Ochrobactrum* genus have been reported to degrade various xenobiotics such as vinyl chloride [26], chlorothalonil [27], [28], methyl parathion [29] dimethyl formamide [30] and nicotine [31]. However, this is the first report of *Ochrobactrum* sp. degrading acetamiprid. *Ochrobactrum* are ubiquitous and numerous in soil and able to survive under extremely harsh conditions. These features make them ideal candidates for bioremediation of contaminated environments.

**Table 2:** Morphological and physio-biochemical characteristics of *Ochrobactrum* sp. D-12.

Characteristics	Results	Characteristics	Results
Colonies	white to beige, mucoid with entire edges	Cells	2–3 mm in diameter within 24 h
Gram-staining	−	β-galactosidase	+
Growth at 42°C	+	α-Glucosidase	+
Growth at pH 9	+	Lipase C14	+
Growth with 7% NaCl	+	Glucose	+
Nitrate reduction	+	Citrate (24 h)	−
indole	−	D-Mannose	+
Aesculin hydrolysis	+	L-Arabinose	−
oxidase	+	D-Turanose	+
catalase	+	L-Lyxose	+
Urease (48 h)	−	Sodium acetate	+

+, tested positive/utilized as substrate; 2, tested negative/not utilized as substrate.

### The pathway of acetamiprid biodegradation by strain D-12

**Figure 2:**
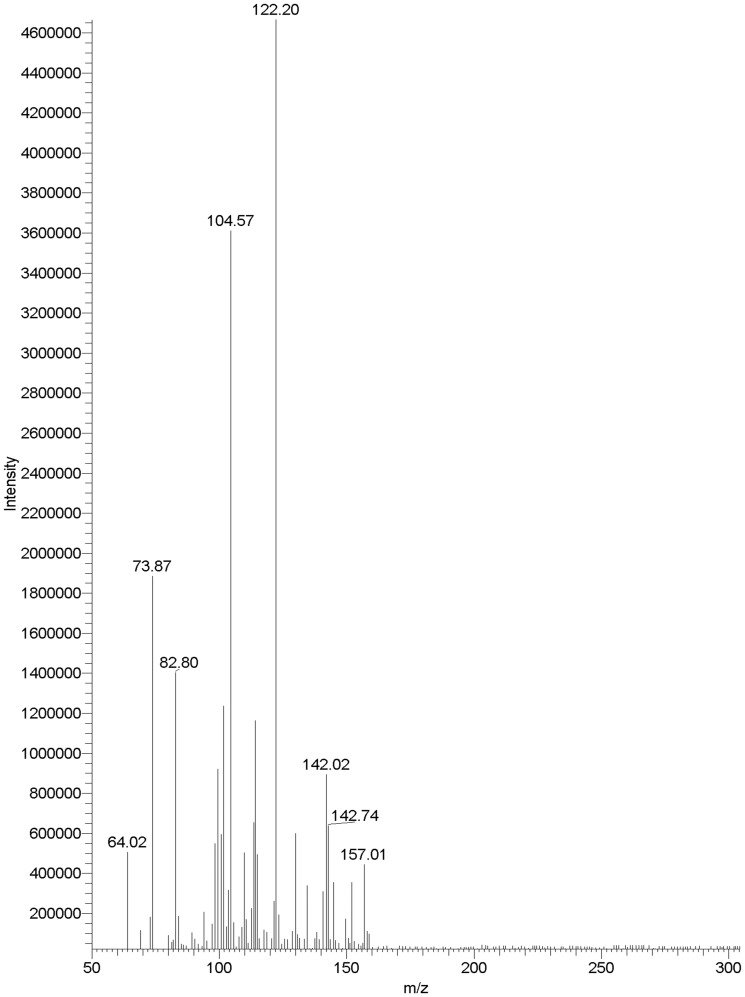
Mass spectrum of the metabolite.

**Figure 3.The:**
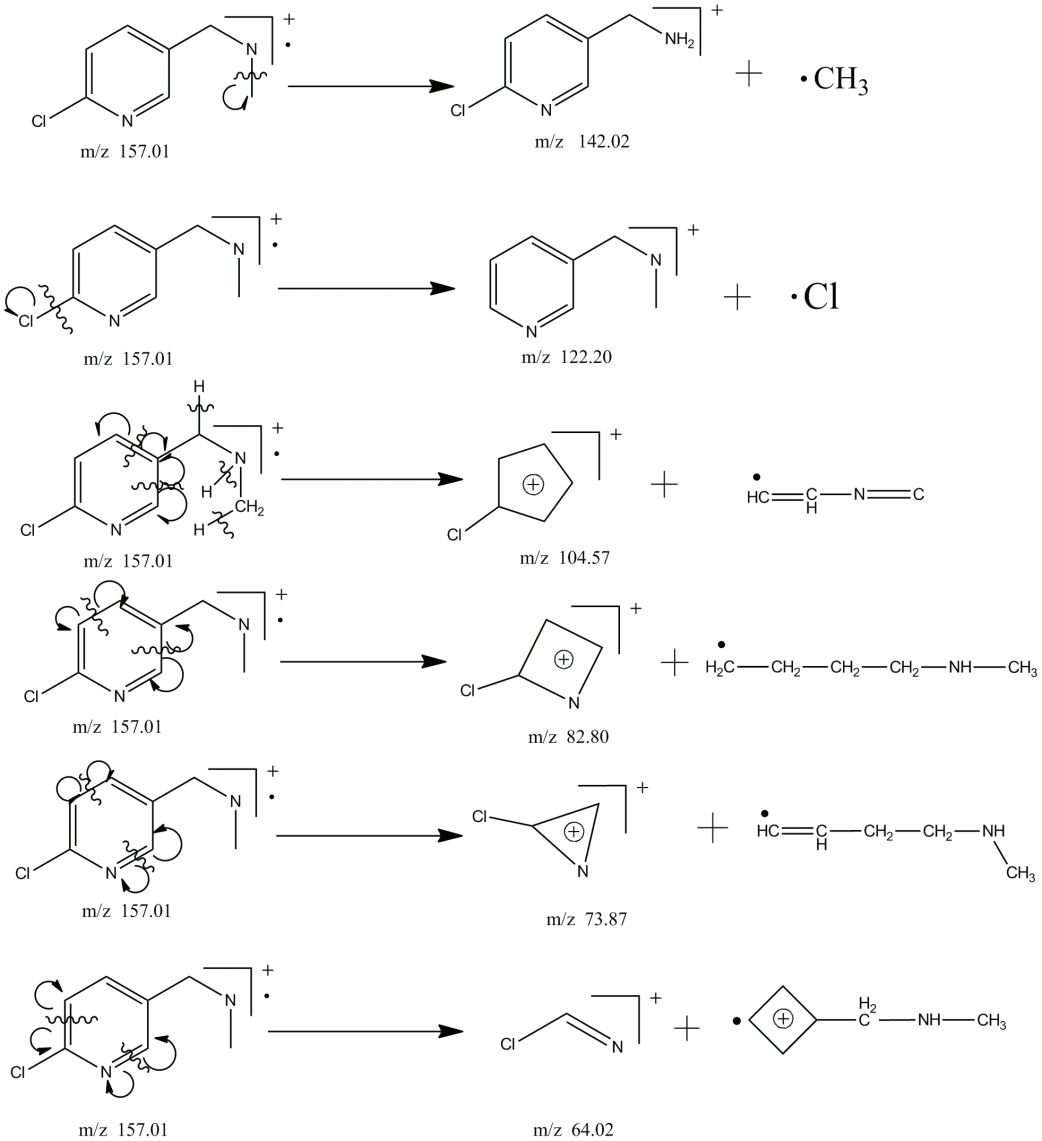
detailed explanation for the MS fragment ion peaks.

As shown in Fig. S2, the metabolite was detected in the HPLC analysis of a 10-day MM culture fluid inoculated with *Ochrobactrum* sp. strain D-12. To determine the structure of the metabolite produced during the degradation of acetamiprid, 15-day cultures of *Ochrobactrum* sp. strain D-12 in MM medium supplemented with 100 µM acetamiprid were subjected to TLC and HPLC. The purified metabolite with the characteristic second-order MS fragment ion peaks at m/z 64.02, 73.87, 82.80,104.57,122.20,142.02 and 157.01 (Fig. 2–3), was identified as *N*-methyl-(6-chloro-3-pyridyl)methylamine. Therefore, the degradation pathway of acetamiprid by isolated D-12 was proposed (Fig. S3). The metabolite idenfied is known to be less toxic to mammals and bees, which was previously identified in mice and honeybees as IM1-4 [32]–[34]. The hydrolytic mechanism is similar to the metabolic conversion of the compound in mammals and insects [13].

### Effects of pH, temperature and initial concentrations on acetamiprid degradation by D-12

The effects of pH and temperature on the aerobic degradation of D-12 in the culture medium were tested after incubation 48 h. From the analysis,we found that pH has significant influence (*P*<0.05) on the experiment by SPSS software(Version 19.0). when the pH was between 6.0 and 8.0, more than 95% of 300 mg·L^−1^ acetamiprid could be degraded by D-12 within 16 h. At pH 7.0, acetamiprid was completely degraded in 14 h. However, when the pH was at 4.0, 5.0, 9.0 or 10.0, acetamiprid biodegradation was distinctly inhibited, implying that restricted growth of D-12 occurred at these pH values. But the temperature has no significant (*P*>0.05).Experiment showed that the optimum temperature for the biodegradation of acetamiprid was 25–35°C. Acetamiprid biodegradation efficiency was the highest at this temperature range; however, acetamiprid biodegradation decreased when the temperature dropped to 20°C or rose to 40°C, indicating that lower and higher temperatures were not beneficial for the biodegradation of acetamiprid by D-12. Therefore, pH 7.0 and 30°C were chosen for all subsequent experiments. At the same time, control experiments were carried out under the same conditions without bacteria. No obvious degradation was detected in the control experiments. The recovery rates of acetamiprid after sample pretreatment were measured between 94% and 102%.

**Figure 4:**
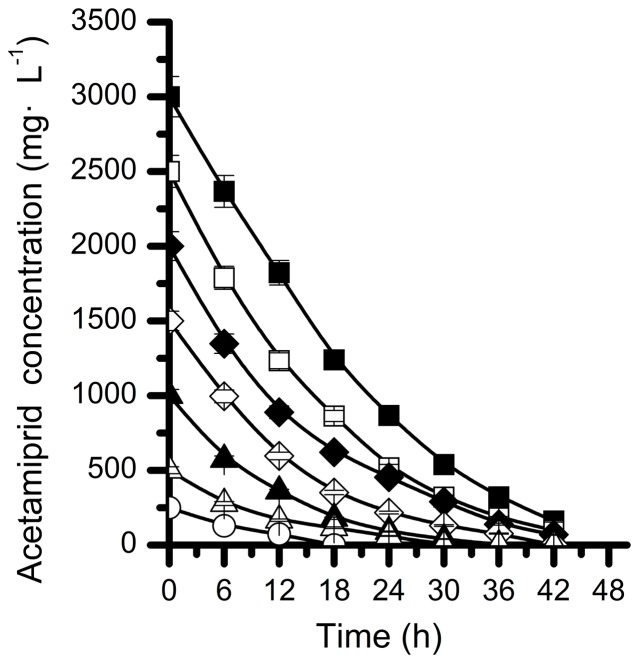
Degradation kinetics of acetamiprid at different initial concentrations by strain D-12. (▪), 3000 mg·L^−1^; (□), 2500 mg·L^−1^; (♦), 2000 mg·L^−1^; (◊), 1500 mg·L^−1^; (▴), 1000 mg·L^−1^; (Δ), 500 mg·L^−1^; (○), 250 mg·L^−1^; values are the means of three replicates with standard deviation.

In order to determine the effect of initial acetamiprid concentrations on degrading efficiency, biodegradation of acetamiprid by *Ochrobactrum* sp. D-12 was conducted under acetamiprid concentrations ranging 250, 500, 1000, 1500, 2000, 2500 and 3000 mg·L^−1^. Strain D-12 grew on acetamiprid up to the concentration, as high as 3000 mg·L^−1^. As shown in Fig. 4, the lag phase was extended at higher acetamiprid concentration. At concentrations of 250, 500, 1000, 1500 and 2000 mg·L^−1^, the degradation rates reached 70.80%, 66.80%, 64.00%, 60.27% and 55.60% after 12 hours of incubation, respectively. However, only 50.68% and 39.27% degradation were achieved at higher initial concentrations of 2500 and 3000 mg·L^−1^, respectively.

The decrease in the specific acetamiprid degradation rate with an increase in the initial acetamiprid concentration suggests that acetamiprid may act as a partial inhibitor to strain D-12. Therefore, the substrate inhibition model Eq. (1) adapted from [35] was used to explain the degradation kinetics of acetamiprid by strain D-12.
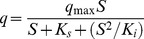
(1)where *q*_max_ is the maximum specific acetamiprid degradation rate (6 h)^−1^, *K*_i_ is the substrate inhibition constant (mg·L^−1^), *K*_s_ is the half-saturation constant (mg·L^−1^), *S* is the substrate concentration (mg·L^−1^), and *S*_m_ is a critical inhibitor concentration of the substrate which decreases degradation.

**Figure 5:**
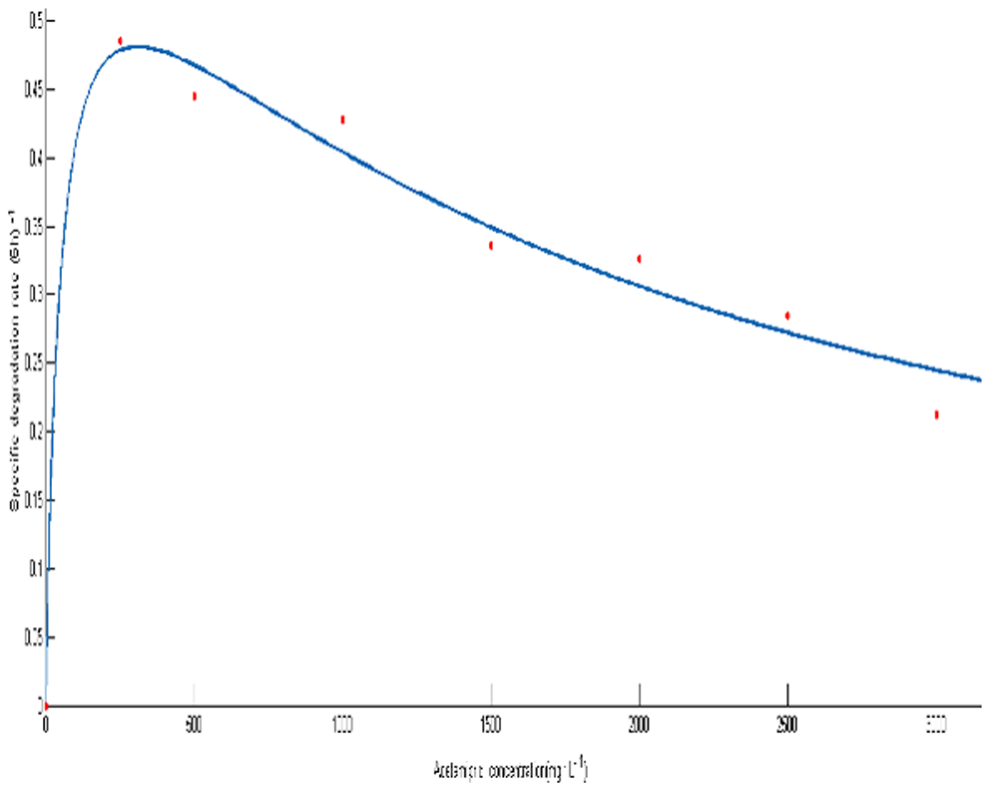
Relationship between specific degradation rate and initial acetamiprid concentration by strain D-12.

The relationship between *q* and initial acetamiprid concentration is shown in Fig. 5. The kinetic parameters of strain D-12 estimated from non-linear regression analysis using matrix laboratory (MATLAB) software (Version 7.8) were *q*_max_ = 0.6394 (6 h)^−1^, *K*_s_ = 50.96 mg·L^−1^, and *K*_i_ = 1879 mg·L^−1^, respectively. The *S*_m_ was established to be 312.8 mg·L^−1^. The value of *R* ^2^ was 0.9827, which indicates that the experimental data were well correlated with the model. As indicated in Fig. 5, when *S* were lower than 312.8 mg·L^−1^, *q* gradually increased. At higher concentrations, inhibition by acetamiprid became prominent.

It was noteworthy that this particular strain could tolerate and efficiently degrade acetamiprid up to the concentration, as high as 3000 mg·L^−1^. However, the specific acetamiprid degradation rate decreased with an increase in the initial acetamiprid concentration (Fig. 5). These findings indicated that increased acetamiprid concentration had a marked effect on degradation performance of strain D-12, but did not lead to complete inhibition. These results proved that strain D-12 was responsible for acetamiprid degradation.

### Effects of different co-substrates on the biodegradation of acetamiprid by strain D-12

**Figure 6:**
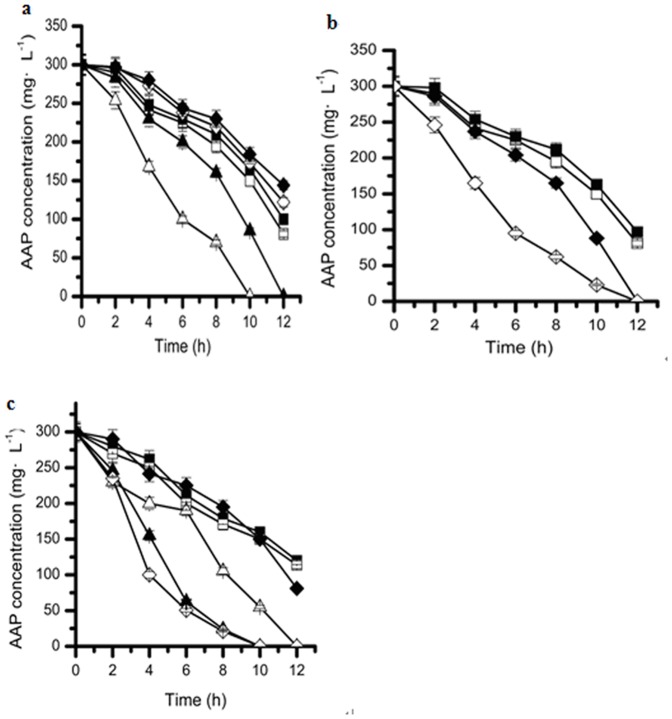
Effect of addition of (a) different concentrations of glucose. (♦), 500 mg·L^−1^; (◊), 400 mg·L^−1^; (▪), 300 mg·L^−1^; (□), control; (▴), 100 mg·L^−1^; (Δ), 200 mg·L^−1^. (b) different concentrations of ammonium chloride. (▪), 900 mg·L^−1^; (□), control; (♦), 600 mg·L^−1^; (◊), 300 mg·L^−1^ (c) different concentrations of glucose with the presence of 300 mg·L^−1^ ammonium chloride as co-substrates on the biodegradation of acetamiprid by strain D-12. (♦), control; (▪), 500 mg·L^−1^; (□), 400 mg·L^−1^; (Δ), 300 mg·L^−1^; (▴), 200 mg·L^−1^; (◊), 100 mg·L^−1^. Error bars, mean ± SD of three replicates.

Industrial wastes often contain a mixture of recalcitrant compounds as well as easily biodegradable compounds. Investigation of the biodegradation of refractory pollutants in the presence of accessible carbon and nitrogen sources might aid in reducing the toxic and growth-inhibiting effects of xenobiotics on cells, thereby increasing the transformation rates of xenobiotics [36]–[38]. However, the reverse consequences had been also reported [39]–[42]. Consequently, effect of the two substrates on acetamiprid biodegradation by strainD-12 was still investigated. Generally, for the bacteria, glucose and ammonium chloride are accessible carbon and nitrogen source, respectively. As shown in Fig. 6 the effect of glucose as a second carbon source or/and ammonium chloride as a second nitrogen source (co-substrate) on the biodegradation of acetamiprid by strain D-12 was investigated.

The acetamiprid degradation efficiency could reach as high as 73% within 12 h when only acetamiprid was added as a sole carbon source (control). Comparatively, after adding 100 mg·L^−1^ glucose as the second carbon source (Fig. 6a), the acetamiprid degradation efficiency was enhanced and 200 mg·L^−1^ acetamiprid was completely degraded within 10 h. However, when more than 300 mg·L^−1^ glucose was added, the acetamiprid degradation efficiency decreased slightly within the same period (Fig. 6a). It has been reported that the addition of glucose promotes the growth of the strain and thus stimulates the degradation of piperazine [43]. The same phenomenon could be observed in the literatures [36]–[38], [44], [45].

It was found that the addition of 300 mg·L^−1^ and 600 mg·L^−1^ ammonium chloride could evidently enhance the degradation process and acetamiprid was completely degraded in less 12 h than the control (Fig. 6b), but even more addition (above 900 mg·L^−1^) of ammonium chloride would delay the biodegradation of acetamiprid by strain D-12. These results are consistent with those of Wang et al. (2012) who found that the addition of 500 mg·L^−1^ ammonium chloride could enhance the biodegradation of 600 mg·L^−1^ nitrobenzene, while the addition of greater amounts of ammonium chloride delayed biodegradation[4]. Luo et al. (2008) founded that the degradation of 100 mg·L^−1^ bensulphuron-methyl in the presence of 1000 mg·L^−1^ ammonium chloride was greater than in the sample without it. Moreover [37], Qiao and Wang (2010), demonstrated the opposite effect, who found that 100 mg·L^−1^ ammonium chloride mildly inhibits the biodegradation of 900 mg·L^−1^ pyridine [46]; Cai et al. (2013) illustrated that the addition of ammonium did not have an evident growth-promoting effect while slightly inhibiting the degradation of piperazine [43]. Based on these studies, appropriate amounts of an extra nitrogen source may inhibited the biodegradation of toxic compounds.

Furthermore, when both glucose and ammonium chloride were added as co-substrates, the degradation efficiency of acetamiprid was much higher compared to that with addition of either glucose or ammonium chloride as carbon or nitrogen source. As shown in Fig. 6a and Fig. 6c, when adding both 100 mg·L^−1^ glucose and 300 mg·L^−1^ ammonium chloride, the acetamiprid degradation efficiency was enhanced by 20% or 50% than that with the addition of same dosage of glucose or ammonium chloride within 10 h, which implied that the performance of biodegradation is likely to be even better in the presence of both carbon and nitrogen sources.

## Conclusions

Strain D-12 isolated in the present study appeared to be highly efficient in degrading acetamiprid in different contaminated soils and water resources, thus suggesting the isolate could be a significant potential use for the cleanup of acetamiprid-contaminated soil. Degradation of acetamiprid occurred at 25–35°C and pH 6–8. This is an important feature of a microorganism to be employed for bioremediation of variable environments. Another important feature which is worth mentioning is that the bacterium utilized acetamiprid as the sole carbon source as well as energy, nitrogen source for growth. Moreover, the strain harbors the metabolic pathway for the detoxification of acetamiprid. Finally, this is the first report about biodegradation of acetamiprid by a bacterial strain from the *Ochrobactrum* genus.

## Supporting Information

Figure S1 **Transmission electron micrograph of strain D-12.** Bar, 1.0 µm.
(TIF)

Figure S2 **The degradation products of acetamiprid in the culture extracts were detected by HPLC.**
(TIF)

Figure S3 **Proposed pathway for acetamiprid degradation by** ***Ochrobactrum*** **sp. strain D-12.**
(TIF)
