# Supplementary figures and images for: Microbial Degradation of Acetamiprid by Ochrobactrum sp. D-12 Isolated from Contaminated Soil
Source: PLoS One. 2013 Dec 27;8(12):e82603. doi: 10.1371/journal.pone.0082603 (PMC3873909; doi:10.1371/journal.pone.0082603)

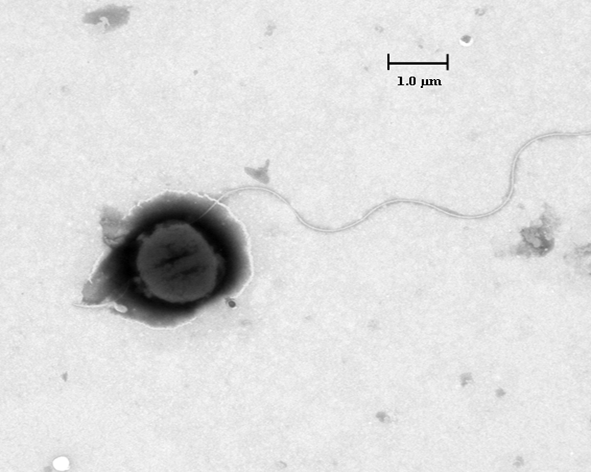

Supplement: Figure S1 — Transmission electron micrograph of strain D-12. Bar, 1.0 µm. (TIF) [file pone.0082603.s001.tif]

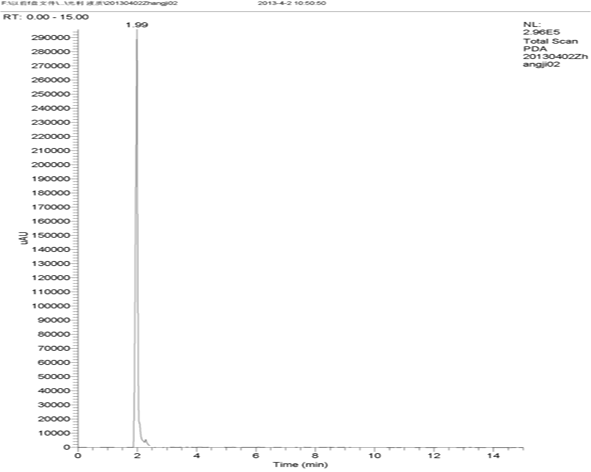

Supplement: Figure S2 — The degradation products of acetamiprid in the culture extracts were detected by HPLC. (TIF) [file pone.0082603.s002.tif]
